# Extracorporeal Removal of Myoglobin in Patients with Rhabdomyolysis and Acute Kidney Injury: Comparison of High and Medium Cut-Off Membrane and an Adsorber Cartridge

**DOI:** 10.1159/000521923

**Published:** 2022-03-25

**Authors:** Alexander Jerman, Milena Andonova, Vanja Persic, Jakob Gubensek

**Affiliations:** ^a^Department of Nephrology, Center for Acute and Complicated Dialysis, University Medical Center Ljubljana, Ljubljana, Slovenia; ^b^Faculty of Medicine, University of Ljubljana, Ljubljana, Slovenia

**Keywords:** Blood purification, Rhabdomyolysis, Myoglobin, Dialysis, Acute kidney injury, CytoSorb

## Abstract

**Introduction:**

The role of extracorporeal myoglobin removal in the treatment of rhabdomyolysis-associated severe acute kidney injury (AKI) is not yet fully established. High cut-off (HCO) and medium cut-off (MCO) dialysis membrane and cytokine adsorber (CytoSorb®) have been used to this purpose in clinical practice. The data on comparative effectiveness of those methods are scarce.

**Methods:**

In this single-center retrospective study, we included patients with AKI and concomitant rhabdomyolysis (myoglobin >20,000 μg/L), who underwent at least one extracorporeal myoglobin removal procedure. The main outcome parameter was myoglobin reduction ratio, whereas albumin was assessed as a safety parameter.

**Results:**

We analyzed data for 15 patients, who underwent 28 procedures (13 HCO, 9 MCO, and 6 adsorber). Pre-treatment serum myoglobin levels were similar between the groups and myoglobin reduction was significant in HCO (*p* = 0.03) and MCO groups (*p* < 0.01) and borderline significant in adsorber group (*p* = 0.06). Reduction ratios were comparable between the groups (median 0.64 (inter-quartile range IQR 0.13–0.72), 0.54 (IQR 0.51–0.61) and 0.50 (IQR 0.37–0.62), respectively, *p* = 0.83). Both pre- and post-procedure serum albumin levels were significantly lower in the MCO group. However, with routine albumin substitution in the HCO group only, serum albumin remained stable during the procedures in all subgroups.

**Conclusions:**

Novel MCO membrane might represent the optimal mode of treatment of severe rhabdomyolysis-associated AKI, as it allows for efficient removal of myoglobin, avoids albumin supplementation and is associated with lower costs. For patients requiring cytokine removal, the adsorption capsule can simultaneously reduce cytokine and myoglobin levels.

## Introduction

Acute kidney injury (AKI) [1] is the most common complication of rhabdomyolysis, occurring in more than half of patients with severe rhabdomyolysis and requiring hemodialysis in about 10–20% [2, 3]. Rhabdomyolysis can result from trauma or crush injury, ischemia, seizures or strenuous exercise, hyperthermia, prolonged immobilization, drug abuse, or medication toxicity. Rhabdomyolysis-associated AKI occurs more often in patients who also have other concomitant risk factors for AKI (e.g., dehydration, sepsis, acidosis). The mechanisms of rhabdomyolysis associated AKI include vasoconstriction, tubular ischemia/injury, tubular obstruction due to myoglobin precipitation [4], and inflammation [5]. Myoglobin half-life is greatly dependent on renal excretion and metabolism and is therefore significantly prolonged in patients with significant AKI, which further aggravates its toxicity. A correlation between high myoglobin levels and the risk for subsequent AKI and need for dialysis was shown [6]. Standard treatment for rhabdomyolysis includes volume resuscitation and urine alkalization, aiming to improve kidney perfusion and excretion of myoglobin. But in cases of significant AKI, myoglobin excretion by kidneys is severely reduced. Given its molecular size (18 kDa), extracorporeal removal of myoglobin is achievable with newer hemodialysis membranes and adsorption techniques. Hemodialysis with high cut-off (HCO) membranes effectively reduces myoglobin levels [7, 8], but there is significant loss of albumin, requiring substitution [9]. Medium cut-off (MCO) membranes are also able to filter myoglobin with minor albumin loss [10], providing stable albumin levels over prolonged periods of use [11]. Hemadsorption with highly porous polymer adsorber is an extracorporeal blood purification technique primarily aimed at removing cytokines and achieving immunomodulation. The adsorption of molecules up to 55 kDa in size is not selective, therefore it also removes myoglobin [12] and due to a very large surface area the adsorber is not saturated quickly. Therefore, hemadsorption can also be used in cases of rhabdomyolysis with severe AKI in the context of sepsis and multi-organ failure [13].

The role of extracorporeal removal of myoglobin in the treatment of rhabdomyolysis-associated severe AKI is not yet fully established. Nevertheless, it is often used in clinical practice, but data on the effectiveness of available methods and especially their comparison are scarce. The aim of our study was to compare the efficacy of three available techniques of extracorporeal myoglobin removal.

## Methods

### Study Design and Data Acquisition

In this single-center retrospective study, we examined medical records of patients treated with hemodialysis or hemoadsorption at our center from April 2018 to August 2020 and included hemodialysis/adsorption procedures fulfilling the following criteria: (a) patients had AKI stage 2–3 by KDIGO definition [1] prior to starting hemodialysis, (b) concomitant rhabdomyolysis with severely increased myoglobin (>20,000 μg/L), (c) the process of rhabdomyolysis was presumably controlled (i.e., embolectomy in case of arterial thromboembolism, rhabdomyolysis occurring after aortic surgery, discontinuation of the offending medication, etc.), (d) the patient underwent at least one hemodialysis procedure with the capability of extracorporeal myoglobin removal (either HCO or MCO membrane or hemoadsorption with CytoSorb[R] adsorber), and (e) laboratory data were available pre- and post-procedure within the specified time-frame (see below). The study was approved by the National Medical Ethics Committee (approval No. 0120-538/2020/3) and written informed consent was waived due to retrospective and observational nature of the study. Patients were identified using existing medical records and data were collected regarding basic epidemiological data, type of the procedure used (HCO, MCO, adsorption with CytoSorb®), treatment duration, blood flow, as well as serum myoglobin, albumin and creatinine before and after each procedure. Data for laboratory values before and after procedure were collected from the time frame of up to 12 h prior to and up to 12 h after the index procedure, reflecting the everyday clinical practice. Serum myoglobin concentrations were measured in our hospital's laboratory using a direct immunochemical chemiluminescence method. The main outcome parameter was reduction ratio for myoglobin which was calculated as RR = (pre − post)/pre (pre − serum myoglobin level before the procedure, post − serum myoglobin level after procedure). Absolute reduction in myoglobin levels was also recorded. Albumin levels before and after the procedure were compared between the groups as a safety parameter.

### Dialysis Techniques

Intermittent dialysis with HCO membrane (Theralite®; Gambro Dialysatoren, Hechingen, Germany) was performed in hemodiafiltration mode with blood flow of 300 mL/min and post-dilution replacement of 3 L/h, usually for 6–8 h duration. Patients treated with HCO membrane were routinely given 200 mL of 20% albumin in the last part of the procedure, according to our HCO dialysis protocol [8]. Intermittent hemodialysis with MCO membrane (Theranova®; Gambro Dialysatoren, Hechingen, Germany) was performed with blood flow of 250 mL/min, for 4–6 h duration and without albumin replacement. Hemadsorption with CytoSorb® cartridge (CytoSorbents Europe, Berlin, Germany) was coupled with either intermittent dialysis or continuous dialysis techniques, and the procedures were performed for about 12 h.

### Statistical Analysis

Data are presented as means ± standard deviation or median and interquartile range, as appropriate. The myoglobin reduction (absolute reduction and reduction ratio) was compared between the three groups with ANOVA and Kruskal-Wallis rank sum test for normally and non-normally distributed parameters, respectively. Differences before and after procedures (within-group) were compared with Student's *t* test and Wilcoxon test for normally and non-normally distributed parameters, respectively. A *p* value of <0.05 was considered statistically significant. Statistical analysis was performed with R version 4.0.3 (2020 The R Foundation for Statistical Computing).

## Results

**Fig. 1:**
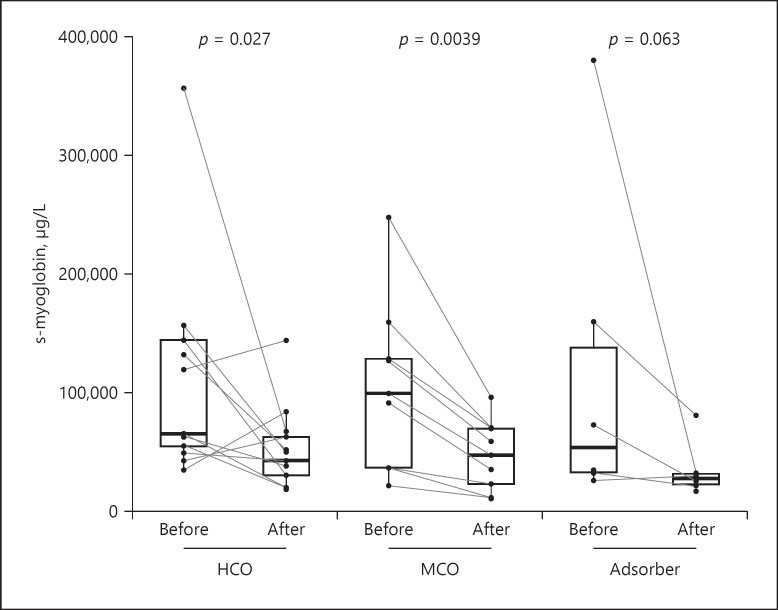
Changes in serum myoglobin by procedure type. Values before and after procedure are compared with Wilcoxon test. For between-group comparison, see Table 2. HCO, high cut-off; MCO, medium cut-off.

Fifteen patients were included (see Table 1) and underwent 28 dialysis procedures: 13 with HCO membrane, 9 with MCO membrane, and 6 with adsorber cartridge. Each of the patients was treated with only one of the before-mentioned type of dialysis/adsorption procedures. Operational parameters of dialysis procedures are presented in Table 2. There were significant differences in treatment duration and blood flow, which resulted from the prescription of the procedures. Pre-treatment serum levels of myoglobin were similar between the groups. Reductions of myoglobin during procedures in all three groups are summarized in Table 2 and presented in Figure 1. The reduction was significant in the HCO and MCO groups and borderline significant in the adsorber group (*p* = 0.06), where the number of procedures was the lowest (*N* = 6). Reduction ratios for myoglobin were comparable in all groups (see Table 2). Both pre- and post-procedure serum albumin were significantly lower in the MCO group (*p* = 0.01 vs. HCO group). With routine albumin substitution in the HCO group only, serum albumin remained stable during the procedures in all subgroups.

## Discussion

In our small retrospective study, we have shown a comparable effectiveness of a newer MCO membrane and a “standard” HCO membrane for myoglobin reduction in patients with rhabdomyolysis and severe AKI. Additionally, contrary to the HCO membrane, there was no need for albumin replacement with the MCO membrane. Cytokine adsorber cartridge also showed comparable effectiveness in terms of myoglobin reduction ratios, although with borderline statistical significance, likely due to small number of procedures. Hemadsorption procedures were also longer, due to their primary indication − cytokine removal.

Extracorporeal myoglobin removal is often considered in clinical practice when the level of serum myoglobin is very high (often >20,000 μg/L) [6] and there is concomitant severe AKI, significantly reducing removal by native kidneys. In light of the pathogenic role of myoglobin, reducing exposure of the kidneys to myoglobin may improve renal recovery and patient outcome. Furthermore, extremely high myoglobin levels cause oxidative stress and possibly systemic toxicity [14, 15]. Although the role of extracorporeal myoglobin removal in severe AKI in terms of improved kidney outcomes has yet to be confirmed in prospective clinical trials, it is nevertheless often used in clinical practice [8, 13, 16]. HCO membranes are efficient in myoglobin removal when used with hemodialysis or hemodiafiltration and were until recently considered as “standard” treatment [8, 16]. But they are associated with significant albumin loss, requiring substitution, and therefore making the procedure more cumbersome and costly [8, 9].

Our study provides preliminary indication that newer MCO membranes probably have comparable effectiveness regrading myoglobin reduction, but without the need for albumin replacement, which is a significant improvement. The results and statistical comparison should be viewed as preliminary given the small number of included procedures and cannot provide definitive evidence for non-inferiority of MCO membranes or the adsorber. Although there is considerable variability within the data, likely reflecting the difference in myoglobin production and removal thought the native kidneys, the absolute values of reduction ratios are quite similar in all groups and their difference between the groups are small and therefore do not suggest clinically significant differences between the compared myoglobin removal methods. In addition to the alleviation of the cost of albumin replacement, MCO dialyzer itself is also significantly cheaper than HCO dialyzer. Cytokine adsorber CytoSorb® is also an attractive option for patients with rhabdomyolysis and concomitant sepsis or other over-inflammation states, enabling concomitant achievement of two treatment goals. However, patients treated with CytoSorb® comprise a specific group: hemodynamically unstable, usually septic patients, who were treated with continuous dialysis methods. Therefore, direct comparison with HCO or MCO membrane dialysis is not possible. In theory, adsorber saturation could also be a problem, contrary to theoretically unlimited removal with diffusion/convection during dialysis. Nevertheless, we have been able to show good efficacy of hemadsorption for myoglobin removal, adding further evidence for its use after initial case reports [12] and case series [13].

The main advantage of our study is a comparison of the effectiveness of the three main methods available in today's clinical practice for myoglobin removal from the blood. Limitations of our study include retrospective design, small number of included procedures, repeated procedures in the same patient, and relatively wide time-frame for laboratory measurements (before and after procedure), which nevertheless reflect real-world clinical decision making, lack of control group (not treated with extracorporeal myoglobin removal), and absence of hard clinical outcomes. The results should therefore be viewed as preliminary and require confirmation in larger cohorts or prospective studies.

## Conclusions

Our preliminary results from a small cohort of patients show that the MCO membrane, as a novel approach, seems to allow for efficient removal of myoglobin from the circulation, comparable to the HCO membrane, but associated with much lower costs and no need for albumin supplementation. Therefore, MCO dialysis might be the optimal mode of treatment of severe rhabdomyolysis-associated AKI. For patients with multiorgan failure requiring cytokine removal, hemadsorption can achieve both goals simultaneously, probably with comparable effect on myoglobin reduction. These results should be confirmed in larger cohorts and prospective studies.

## Statement of Ethics

The study was approved by the National Medical Ethics Committee (No. 0120-538/2020/3) and written informed consent was waived due to retrospective and observational nature of the study.

## Conflict of Interest Statement

J.G. reports receiving reimbursements and speaking honoraria from Baxter. Other authors have no conflicts of interest to declare.

## Funding Sources

The authors acknowledge the financial support from the Slovenian Research Agency (research core funding No. P3-0323) and the University Medical Center Ljubljana (Research & Development Grant No. 20200207).

## Author Contributions

J.G. and V.P. designed the study. M.A. and A.J. collected data. A.J. performed statistical analysis and wrote the draft. J.G., V.P., M.A., and A.J. interpreted the results. All authors contributed significantly to the manuscript and have read and approved the final version.

## Data Availability Statement

The datasets used and/or analyzed during the current study are available from the corresponding author upon reasonable request.

## Figures and Tables

**Table 1:** Basic characteristics of the included patients (*n* = 15)

Female	2 (13)
Age, years	58.8±15.2
Cause of rhabdomyolysis	
Limb ischemia	5 (33)
Medication induced	5 (33)
Crush syndrome	1 (7)
Other	4 (27)
Creatinine before 1st procedure, µmol/L	292±136
Myoglobin before 1st procedure, µmol/L	65,320 [35,511–159,348]
Sepsis	6 (40)
Admitted to ICU	13 (87)
Mechanical ventilation	11 (73)

Data are presented as frequency (percentage), mean ± standard deviation or median [inter-quartile range]. ICU, intensive care unit.

**Table 2:** Operational parameters of dialysis procedures and laboratory results for all three groups

Parameter	HCO	MCO	Adsorber	*p* value
*N*	13	9	6	−
Treatment duration, h	8 [6–8]	5 [4–6]	11 [10–12]	<0.001
Blood flow, mL/min	300 [300–300]	250 [250–250]	250 [250–250]	<0.001
Dialysis modality	HDF 13 (100)	HD 9 (100)	CVVHD 1 (17) HD 5 (83)	−
Pre-procedure s-myoglobin, µmol/L	65,320 [54,931–143,999]	99,379 [36,624–128,491]	53,646 [32,731–137,828]	0.82
Post-procedure s-myoglobin, µmol/L	42,849 [30,163–62,600]	47,034 [23,010–69,639]	27,583 [22,550–31,491]	0.49
Before/after comparison	*p* = 0.03	*p* = 0.004	*p* = 0.06	−
Myoglobin decrease, µmol/L	42,959 [6,539–10,6734]	56,226 [24,638–68,096]	32,554 [12,268–70,962]	0.80
Myoglobin reduction rate	0.64 [0.13–0.72]	0.54 [0.51–0.61]	0.50 [0.37–0.62]	0.83
Albumin before, g/L	31±3	27±3	28±3	0.03
Albumin after, g/L	32±3	28±33	28±5	0.03
Before/after comparison	*p* = 0.56	*p* = 0.41	*p* = 0.81	−

Data are presented as frequency (percentage), mean ± standard deviation or median [inter-quartile range]. HDF, hemodiafiltration; HD, hemodialysis; CVVHD, continuous hemodialysis.
